# MEPHAS: an interactive graphical user interface for medical and pharmaceutical statistical analysis with R and *Shiny*

**DOI:** 10.1186/s12859-020-3494-x

**Published:** 2020-05-11

**Authors:** Yi Zhou, Siu-wai Leung, Shosuke Mizutani, Tatsuya Takagi, Yu-Shi Tian

**Affiliations:** 1 Graduate School of Pharmaceutical Sciences, Osaka University, 1-6 Yamadaoka, Suita City, Osaka, 565-0871 Japan; 2 State Key Laboratory of Quality Research in Chinese Medicine, Institute of Chinese Medical Sciences, University of Macau, Macao, China; 3 School of Informatics, College of Science and Engineering, University of Edinburgh, Edinburgh, UK

**Keywords:** Medical statistics, Data analysis, Statistical software, Partial least squares

## Abstract

**Background:**

Even though R is one of the most commonly used statistical computing environments, it lacks a graphical user interface (GUI) that appeals to students, researchers, lecturers, and practitioners in medicine and pharmacy for conducting standard data analytics. Current GUIs built on top of R, such as EZR and R-Commander, aim to facilitate R coding and visualization, but most of the functionalities are still accessed through a command-line interface (CLI). To assist practitioners of medicine and pharmacy and researchers to run most routines in fundamental statistical analysis, we developed an interactive GUI; i.e., MEPHAS, to support various web-based systems that are accessible from laptops, workstations, or tablets, under Windows, macOS (and IOS), or Linux. In addition to fundamental statistical analysis, advanced statistics such as the extended Cox regression and dimensional analyses including partial least squares regression (PLS-R) and sparse partial least squares regression (SPLS-R), are also available in MEPHAS.

**Results:**

MEPHAS is a web-based GUI (https://alain003.phs.osaka-u.ac.jp/mephas/) that is based on a *shiny* framework. We also created the corresponding R package *mephas* (https://mephas.github.io/). Thus far, MEPHAS has supported four categories of statistics, including probability, hypothesis testing, regression models, and dimensional analyses. Instructions and help menus were accessible during the entire analytical process via the web-based GUI, particularly advanced dimensional data analysis that required much explanation. The GUI was designed to be intuitive for non-technical users to perform various statistical functions, e.g., managing data, customizing plots, setting parameters, and monitoring real-time results, without any R coding from users. All generated graphs can be saved to local machines, and tables can be downloaded as CSV files.

**Conclusion:**

MEPHAS is a free and open-source web-interactive GUI that was designed to support statistical data analyses and prediction for medical and pharmaceutical practitioners and researchers. It enables various medical and pharmaceutical statistical analyses through interactive parameter settings and dynamic visualization of the results.

## Background

Statistical analysis is crucial to research endeavors, but it can be intimidating for medical and pharmaceutical practitioners and students. Few pharmaceutical researchers and practitioners can afford the time and costs to learn how to use commercial statistical tools, such as SAS (https://www.sas.com/), Stata (https://www.stata.com/), and SPSS (https://www.stats-guild.com/ibm-spss), let alone spending time learning statistical computing properly using free and open-source software (FOSS) programs such as R (https://www.r-project.org/) and Python (https://www.python.org/). There is a demand from the medical and pharmaceutical practitioners and researchers for free and accessible graphical user interfaces (GUIs) as well as step-by-step guidance to run data analysis.

As a widely used FOSS environment for statistical computing and visualization, R provides abundant functions to users. However, users are required proficient programming skills and profound knowledge of statistics. The program R-Commander [1] and its plug-in EZR [2] were built on top of R to provide rudimentary GUIs; however, they must be installed on local computers and still need solid knowledge of R programming. Further, the GUIs of EZR and R-Commander are not accessible via smartphones or tablets.

Considering the powerful functions in R, we created a *shiny*-based [3] web application Medical and Pharmaceutical Statistics (MEPHAS). We set up MEPHAS online as a ready-to-use web server (https://alain003.phs.osaka-u.ac.jp/mephas/) for public access. The underlying R functions were created as the R package named *mephas* (https://mephas.github.io). Unlike R-Commander and EZR, the webserver MEPHAS is highly accessible via various computing devices such as laptops, tablets, and smartphones.

MEPHAS was designed to support researchers in medical and pharmaceutical fields and can enable medical and pharmaceutical practitioners and researchers to conduct confirmatory and exploratory data analyses. Through a *shiny* framework**,** MEPAHS provides a web-based interactive GUI that can dynamically visualize real-time analytical results across multiple panels simultaneously. Notably, this analytical mode avoids repetitive and redundant operations when finding optimal parameters, and therefore, the analytical process is facilitated.

MEPHAS provides separate interfaces to access numerous medical and pharmaceutical statistics, and it offers easy-to-follow instructions when necessary so that users can analyze their data without the need to acquire a profound knowledge of statistical analysis beforehand. The methods available in MEPHAS refer to several popular textbooks and critical requests on medical and pharmaceutical statistical analysis. We created probability and hypothesis testing interfaces based on the concepts from the *Fundamentals of Biostatistics* [4]. With reference to *Applied Regression Analysis* [5] *and Other Multivariable Methods* and *Modeling Survival Data* [6]*,* we added regression models for continuous, binary, or time-to-event outcomes. To support the high throughput omics data analysis and quantitative structure-activity relationship (QSAR) models, we included partial least square regression (PLS-R) [7] and sparse partial least square regression (SPLS-R) methods [8]. To fill the voids of existing affordable software that only support basic models on time-to-event outcomes, we added the accelerated failure time (AFT) models [9] and extended Cox regression with random effect terms [6, 10]. Notably, MEPHAS is the first *shiny* application to support the AFT model, PLS-R, and SPLS-R with an interactive GUI with downloadable result tables and figures. Additionally, based on those statistical models, MEPHAS can generate prediction results of the new data.

The objectives of MEPHAS are (1) to guide researchers in using basic and advanced statistical methods in medical and pharmaceutical research; (2) to create an integrated platform for medical and pharmaceutical statistics and fill the void as with the extended Cox regression, AFT model, PLS-R, and SPLS-R; and (3) to facilitate the data analytical process by allowing various interactive operations.

## Implementation

MEPHAS was implemented with various R packages (Additional file 1: AF_list.docx) for statistical operations and the package *shiny* for the web-based GUI. The MEPHAS web server was built on CentOS 7. The R package *mephas* was developed with R (version 3.6.1) and RStudio (version Version 1.2.5001).

### Availability and usage

Users can use either the MEPHAS webserver or install R packages locally according to the following instructions.

The MEPAHS webserver can be accessed at https://alain003.phs.osaka-u.ac.jp/mephas/. It is accessible through most major web browsers with current versions installed in Windows, macOS (iOS), Android, or Linux. Users can use the webserver by referring to the help and tutorials.

The underlying functions of MEPHAS have been wrapped in R package *mephas* and *mephas.tools* for users to install locally. The R package *mephas.tools* contains the subsidiary functions for MEPHAS, while the R package *mephas* includes the services to activate the interfaces. Both packages are published on GitHub (https://mephas.github.io). The installation command is based on the R package *remotes* [11]. After installing *remotes*, users can download and install the latest version from GitHub by typing the following command in R console (**Code 1**).

### Code 1 installation code of R package *mephas* and *mephas.tools* from GitHub

> > install.packages(“remotes”)

> > remotes::install_github(c(“mephas/mephas.tools”,“mephas/mephas”),upgrade = “never”)

Users need to load the functions in the package *mephas.tools* and *mephas* after installation and use function mephasOpen to activate the web-based interfaces (**Code 2**).

### Code 2 function to open MEPHAS interface

> > library(“mephas”).

> > mephasOpen (method = “condist”).

The list of methods and contents of the functions in R package *mephas* can be found in the documentation (https://mephas.github.io/reference/index.html).

The GUI is activated on the users’ default browsers when they use the R console. If users run the codes in RStudio’s console, the GUI is shown in RStudio’s window. Users can minimize the console and use only the web-based GUI to input data, choose parameters, and generate results.

### Design

**Fig. 1:**
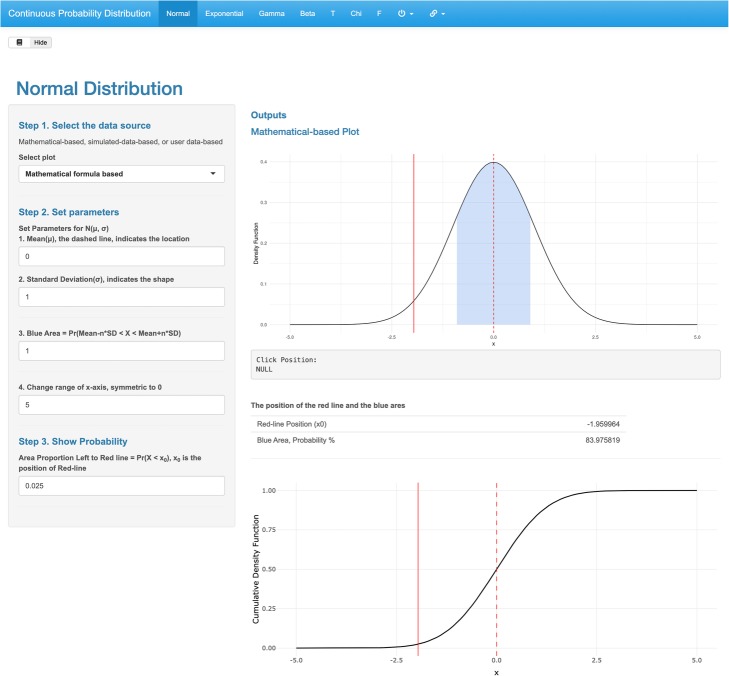
The layout of MEPHAS in the interface of *Continuous Probability Distribution*

To date, MEPHAS has supported four categories of statistical analysis (*probability, hypothesis testing, regression model, and dimensional analysis*) that were developed in 12 independent web-based interfaces (Additional file 1: AF_table1.docx). Every interface has a similar construction and design. There are tabs for different methods, an input panel on the left, and an output panel on the right (Fig. 1). The general functionalities include data input, parameter configuration, and result output.

#### Data input

MEPHAS comes with example data for interpreting purposes. Easy-to-use example data are available in each interface for users to use and practice. Some example data are available in the input box on the left-side panel for users to start with, overwrite, or modify. Some example datasets are embedded in the interface for users to choose. All the data to be used can be previewed in the Data Preview panel.

Except for choosing or overwriting the example data, MEPHAS provides a tab for “Upload CSV file” for users to upload their data from local devices. Users can upload a CSV file by clicking the “Browse … ➔Select data➔ Open” button.

#### Configuration

After input or upload the data, configurations are required for generating the statistical results. The configuration includes setting parameters and preprocessing the data. To facilitate the method for choosing the parameters in statistical analysis, MEPHAS takes advantage of various widgets for users to control the input. Checkbox gives a binary choice; the input box reminds users to input values; the radio buttons and select box help users to make decisions among different situations; the slider bar helps users choose values.

MEPHAS provides some functions to enable users to preprocess the data. For manually input data, users can alter the variable names when necessary. For regression models and dimensional analyses, users can transform the data, change the types of variables from numeric to categorical, and vice versa, and remove some possible outliers. Specifically, the factor level of a categorical variable is changeable for regression models.

#### Results output

MEPHAS provides statistical results via reactive statistical tables and plots. Most statistical results are shown in the responsive tables that are downloadable and enable users to page, filter, search, and sort the values. MEPHAS adopted interactive plots that show the real-time values on the plots and can be configured by users to download. MEPHAS also provides statistical 3D-dimensional visualization to support dimensional analyses.

### Statistics

To help users find the proper statistical methods from the interfaces, MEPHAS provided a flowchart (https://alain003.phs.osaka-u.ac.jp/mephas/MEPHAS_flow.pdf).

#### Probability

Two interfaces, *Continuous Probability Distribution* and *Discrete Probability Distribution,* are designed to provide visualization of the commonly used statistical probability distributions; thus, complex theories are not presented. Users can view how the probability distribution curves and cumulative probability curves are altered when they resize the parameters. This allows them to understand the functionalities of the parameters. Particularly, MEPHAS provides a histogram from the simulated numbers to illustrate the process that simulated numbers approximate to the real distribution. The simulation numbers can be downloaded. Users can also upload new data and generate the distribution plots from new data. Some supplementary results, such as the mean, standard deviation, and the area to the left of the percentage points, are also presented.

#### Hypothesis testing

Hypothesis testing helps users quickly access and master their desired methods. This topic includes five interfaces: i.e., *Parametric T Test for Means, Non-parametric Test for Medians, Test for Binomial Proportions, Test for Contingency Tables,* and *Analysis of Variance.* Under each of these five methods, MEPHAS presents introductions on the methodology, case examples, and necessary explanations. By following the guidance and embedded example data, users can locate a suitable test and obtain the real-time outputs quickly.

#### Regression model

The interfaces for the regression model include *Linear regression*, *Logistic regression,* and *Survival Analysis*. These interfaces separate data preparation from model construction and provide a step-by-step guide in building the statistical models. The first (and compulsory) step is to choose the independent and dependent variables from the prepared dataset. The second step is to remove or keep the addictive terms. For example, whether to add interactive terms and a constant, whether to add the random effect term, such as cluster, strata, and frailty terms, in the Cox regression and AFT model. After the formula of the statistical model is constructed correctly, the third step is to check the formula and click the button to generate results. In the outputs, MEPHAS presented abundant results in parameter estimation and model evaluation. Furthermore, MEPHAS enables users to upload new datasets and derive prediction and evaluation results based on the existed model.

#### Dimensional analysis

The dimensional analysis includes two interfaces. One interface contains the principal components analysis and explanatory factor analysis; the other contains Principal Component Regression (PCR), PLS-R, and SPLS-R. These methods are often used to analyze high-dimensional data, such as gene expression and chemical data. PLS-R and the related method are widely used in QSAR analysis [7]. SPLS-R was developed to select variables as well as derive principal components with application in omics data [8]. Among these methods, the common problem is to decide the optimal number of components. Usually, users need to repetitively change the number of components and determine the optimal scenario according to the results. MEPHAS facilitates this optimization process by using real-time feedback. Users can adjust the parameters on the left-side panel and immediately obtain the results on the right (main)-side panel for decisions about the parameter settings. In detail, we provide all possible choices of algorithms for model fitting and validation to remind users of other possible methods for data analyses. To better visualize the results, MEPHAS provided reactive plots in 2D and 3D dimensions. For PCR, PLS-R, and SPLS-R, MEPHAS enables users to upload new data for prediction.

## Results

We compared MEPHAS with another three open-source software: EZR, Free Statistics and Forecasting Software (FSFS, https://www.wessa.net/stat.wasp), and Radiant - Business analytics using R and Shiny (https://radiant-rstats.github.io/docs/). All of the three software are well-structured and cover the four categories of statistics (*probability, hypothesis testing, regression model, and dimensional analysis*). EZR is R console-based GUI and was designed for the application of statistical functions that are frequently used in clinical studies. FSFS is published on web-server, and the results are generated from R. Radiant is based on the shiny framework and was designed for business data analysis. In the general comparison on the utilities, MEPHAS added prediction functions in the statistical models and facilitate users to upload new data to predict and evaluate the prediction results. MEPHAS provided more reactive tables and plots to illustrate the analytical results. Notably, MEPHAS is the only one that included 3D visualization.

To compare the variety of functions, we listed all the methods implemented in the four software (Additional file 1: AF_table2.docx). In general, MEPHAS and EZR share most methods owing to the similar application filed of medical statistics. In the probability distribution, only MEPHAS, FSFS, and Radiant have the functions to plot probability distributions. MEPHAS has more functions, such as to generate cumulative probability density plot and random numbers. In the hypothesis testing, EZR has most methods while MEPHAS also included many often used ones. EZR and MEPHAS can be supplementary of each other. In the regression model, FSFS and Radiant emphasize on the linear regression and logistic regression, while EZR and MEPHAS have survival analysis that is often used to analyze the time-to-event in cancer data [2]. MEPHAS enables users to analyze left-truncated and right-censored time and the Cox regression with random effect term. The addition of the AFT model in MEPHAS remedies a major defect on most software [12]. Except for analyzing data, MEPHAS has a panel for users to upload new data for real-time prediction, and such functionality was not found in the other three software. Due to the different application background, not all the software have dimensional analysis methods, and MEPHAS also lacks the calculation of sample size and some statistical methods that are not often used for medical or omics data.

Two examples were selected to illustrate the performance of MEPHAS in building the models on medical data and gene expression data.

### Example 1: Acute leukemia dataset

Data downloaded from the EZR website (http://www.jichi.ac.jp/saitama-sct/SaitamaHP.files/sample.csv) includes the time-to-event records of 93 fictional patients who received Allo-SCT for acute leukemia. Time-to-event data include information of both time and censor. Therefore, survival analysis is the optimal choice for modeling.

The first step of building a model is to upload data and prepare the time-to-event survival object. We opened the interface of *Survival Analysis* of MEPHAS, and upload the sample data in the first tab. Because this dataset does not have row names in the first column, we changed the options for row names. The successfully uploaded data were shown on the right-side (Additional file 1: AF_result.docx 1-a). In survival analysis, the most common type of time-to-event data is the right-censored time. The right-censored time requires at least two variables: a numeric time-to-event variable and a binary status variable with 1 indicating death and 0 indicating censor. The next commonly encountered time is left-truncated and right-censored time that requires three variables: a numeric start-time variable, a numeric end-time variable, and a binary status variable; where the end-time should always be greater or larger than the start-time. MEPHAS is capable of handling these two types of survival time. Users are only required to click the correct variables and choose the type of survival time to create the survival objective. In this example, because time is right-censored type, we chose “OS” and “DaysToOS” to generate the survival objective (Additional file 1: AF_result.docx 1-a). After the preparation of data and survival objects, the exploratory analyses would come out, including the basic descriptive statistics, three types of survival curves, life table, and histogram and density of a particular chosen variable (Additional file 1: AF_result.docx 1-b ~ 1-d).

The functions in *Survival Analysis* includes (1) non-parametric model such as Kaplan-Meier method to estimate the survival probability and log-rank test to compare the differences between survival probability from different groups; (2) semi-parameter model such as the Cox regression; and (3) parametric model such as AFT model. We used MEPHAS to generate the same results from EZR in the log-rank test and basic cox regression (Additional file 1: AF_result.docx 2-a ~ 2-c, 3-a ~ 3-e). In MEPHAS, the survival plots are not limited to the survival probability curves, but also the cumulative event and cumulative hazard. MEPHAS also created more residuals plots in an interactive way. Martingale residual and deviance residual plots can help users find the outliers in the data. Cox-snell residual plot assesses the goodness-of-fit of the model. In the model, we chose four independent variables: “Age,” “Source,” “DiseaseRisk,” and “Regimen.” The martingale residuals and deviance residuals found two outliers in red at the bottom of plots. The Cox-snell residual plot indicated that the goodness-of-fit became worse in the later period (Additional file 1: AF_result.docx 3-f).

The prediction panel below enables users to upload a new data set to predict the linear part and the risk scores of the Cox regression (Additional file 1: AF_result.docx 4-a ~ 4-c). To illustrate this function, we split the sample data into a training set and a test set (“sample_train.csv” and “sample_test.csv”). The evaluation methods of Brier score and various time-dependent AUC were also available. Brier score gave a time-dependent prediction error curve [13], and time-dependent AUC gave the AUC at user-defined points. From the evaluation of prediction results, we found that the Brier score increased, and AUC decreased in the later period, indicating the worse prediction ability at the later time.

MEPHAS also added random effect functions that extended the Cox regression in analyzing the effect of heterogeneity in the data. Except for the common strata function, MEPHAS provided three more methods to analyze the heterogeneity: clustering, gamma frailty, and gaussian frailty. In the illustration, we compared the effect of “Relapse” as strata, clustering, and gamma frailty terms. Clustering estimates the relative risks among the “Relapse” group while frailty term estimates the relative risks in the “Relapse” group. In this example, the addition of the random effect terms increased the concordance index from 0.744 (basic Cox regression) to 0.794 (frailty). The frailty model indicated that the hazard of “Relapse = 1” is approximately 4.5 times of “Relapse = 0” (1.6348/0.3652 ≈ 4.48, Additional file 1: AF_result.docx 5-a ~ 5-c).

### Example 2:Yeast cell cycle dataset

The statistical results and plots from PLSR related methods are not easy to understand, and not many web-based software can conduct SPLS-R. We used the yeast cell cycle example [8] to illustrate SPLS-R in analyzing gene expression data. This dataset has a total of 18 measurements covering two cell cycle periods. These 18 measurements are the 18 columns of the response matrix in the dataset. The predictors are chromatin immunoprecipitation on chip data containing the binding information for 106 transcription factors.

The first step was to upload data into the Data tab in *Dimensional Analysis 2*. After the successful upload of data, descriptive statistics would be created automatically. The linear fitting plot allowed users to view the relation between two variables. Users can choose the variables to plot the interactive heatmap with or without scaling the data (Additional file 1: AF_result.docx 6-a ~ 6-c).

We built the model in the SPLS-R tab. We first chose 18 dependent variables, and the other independent variables were thrown in the independent variable box automatically. Before inputting the parameters, we conducted cross-validation to decide the optimal parameters K from the range of [1, 10] and eta from the range of [0.1, 0.9], where K is the number of components and eta controls the selection range. Cross-validation showed that the optimal parameters were K = 8 and eta = 0.6. Thus we used these parameters in the model, and SPLS-R selected 50 variables. The selected variables could be downloaded if needed.

MEPHAS generated various reactive tables and plots for view and download. Except for the coefficient path plot available in the original method, MEPHAS created a more reactive plot for loadings and scores values that help users to find the outlier sample easily and check the amount of a variable that contributes to a particular component. The 3D visualization plot further helped users to view the distribution of scores and loadings (Additional file 1: AF_result.docx 7-z ~ 7-e).

To illustrate the prediction in SPLS-R, we used the training and test data derived from the yeast cell cycle dataset (“yeast_train.csv” and “yeast_test.csv”). The predicted results could be download without affecting any other parameters or results in the model (Additional file 1: AF_result.docx 8).

## Discussion

We developed an interactive web-based GUI application MEPHAS to facilitate non-trivial statistical analysis for both researchers and doctors in pharmaceutical fields via devices ranging from tablets to workstations. We also implemented an R package *mephas* executable under Windows, macOS, and Linux environments. With the user-friendly GUI available under various computing environments, users can perform data analysis tasks such as managing data, analyzing data, and visualizing the results step by step without intensive statistical software training.

MEPHAS covers adequate pharmaceutical statistics including statistical probability distributions and hypothesis testing with various data types. It also provides advanced statistical methods such as analysis with regression models and dimensional analyses. In addition, MEPHAS extends regression models to cover random or interactive effects and enable the prediction of the new dataset. MEPHAS made up for the lack of the AFT model in most statistical software and provided two types of time-to-event data analysis. It is also the first web-based application to produce dynamic results and 3D plots for PLS-R and SPLS-R methods.

In contrast to the statistical software programs that are sophisticated but operate through a CLI, MEPHAS simplifies all data preprocessing operations with intuitive widgets, such as check and select boxes. Different from the software with static GUI, MEPHAS organizes the input and output panels under multiple tabs to provide rich sets of functionalities under a single integrative and interactive web-based interface. This interactivity enables users to view the real-time output and adjust all parameters during the analytical process, saving much time that was spent in repetitive operations with static GUI panels in other software. Amongst the web-based statistical applications, the majority are isolated and were created for only one analytical objective. MEPHAS integrated the statistical methods for medical, chemical, and omics data analysis and provided the flowchart to help people find the proper methods.

We have been improving MEPHAS throughout many pre-release versions to optimize the GUI design and simplify the operations. MEPHAS has now reached a stable version with robust architecture to accommodate further extensions. A developer can take advantage of MEPHAS’s open-source codes under an MIT license accessible from GitHub to customize the *mephas* package or its web server MEPHAS. Forthcoming versions will mainly provide additional methods, such as predictive models combining PLS and survival analysis associated with patients’ gene expression data and machine learning algorithms for chemoinformatic data mining.

## Conclusions

MEPHAS is a free interactive GUI of R that was developed to support statistical data analyses for medical and pharmaceutical students and practitioners. It is the first integrated web application for statistical analysis that combines various medical and pharmaceutical statistical applications through interactive parameter settings and dynamic visualization of the results.

## Availability and requirements

**Project name:** MEPHAS (Medical and Pharmaceutical Statistics).

**Project home page:** https://mephas.github.io/

**Operating system(s):** Platform independent.

**Programming language:** R.

**Other requirements:** Internet browser.

**License:** MIT.

**Any restrictions to use by non-academics:** None.

## Supplementary information

**Additional file 1: AF_table1.docx** Graphic user interfaces and statistical methods in MEPHAS; **AF_table2.docx** Comparison of methods in MEPHAS with EZR, FSFS, and Radiant; **AF_list.docx** R packages used in MEPHAS; **AF_result.docx** The results in Example 1 and Example 2.

## Data Availability

The datasets are also available online at https://github.com/mephas/datasets/tree/master/csv_in_manuscript.

## Abbreviations

AFT: Accelerated Failure Time
CLI: Command-line Interface
CSV: Comma-separated Values
CV: Cross-validation
FOSS: Free and Open-source Software
FSFS: Free Statistics and Forecasting Software
GUI: Graphical User Interface
MEPAHS: Medical and Pharmaceutical Statistics
PCR: Principal Component Regression
PLS-R: Partial Least Squares Regression
QSAR: Quantitative Structure-Activity Relationship
SPLS-R: Sparse Partial Least Squares Regression
