# Supplementary material for: MEPHAS: an interactive graphical user interface for medical and pharmaceutical statistical analysis with R and Shiny
Source: BMC Bioinformatics. 2020 May 11;21:183. doi: 10.1186/s12859-020-3494-x (PMC7216538; doi:10.1186/s12859-020-3494-x)
Supplement: Supplementary file 1 — Additional file 1: AF_table1.docx Graphic user interfaces and statistical methods in MEPHAS; AF_table2.docx Comparison of methods in MEPHAS with EZR, FSFS, and Radiant; AF_list.docx R packages used in MEPHAS; AF_result.docx The results in Example 1 and Example 2. [file 12859_2020_3494_MOESM1_ESM.zip › AF_list.docx]

**List R packages used in MEPHAS**

| **Package name** | **Link** |
| --- | --- |
| *DescTools* | https://CRAN.R-project.org/package=DescTools |
| *DT* | https://CRAN.R-project.org/package=DT |
| *exactRankTests* | https://CRAN.R-project.org/package=exactRankTests |
| *dunn.test* | https://CRAN.R-project.org/package=dunn.test |
| *ROCR* | https://CRAN.R-project.org/package=ROCR |
| *ggplot2* | https://CRAN.R-project.org/package=ggplot2 |
| *magrittr* | https://CRAN.R-project.org/package=magrittr |
| *psych* | https://CRAN.R-project.org/package=psych |
| *pls* | https://CRAN.R-project.org/package=pls |
| *plotly* | https://CRAN.R-project.org/package=plotly |
| *reshape* | https://CRAN.R-project.org/package=reshape |
| *scales* | https://CRAN.R-project.org/package=scales |
| *shiny* | https://CRAN.R-project.org/package=shiny |
| *shinythemes* | https://CRAN.R-project.org/package=shinythemes |
| *shinyWidgets* | https://CRAN.R-project.org/package=shinyWidgets |
| *survival* | https://CRAN.R-project.org/package=survival |
| *survminer* | https://CRAN.R-project.org/package=survminer |
| *survAUC* | https://CRAN.R-project.org/package=survAUC |
| *spls* | https://CRAN.R-project.org/package=spls |
| *stargazer* | https://CRAN.R-project.org/package=stargazer |
| *stats* | https://CRAN.R-project.org/package=stats |
| *utils* | https://CRAN.R-project.org/package=utils |
