# Supplementary material for: MEPHAS: an interactive graphical user interface for medical and pharmaceutical statistical analysis with R and Shiny
Source: BMC Bioinformatics. 2020 May 11;21:183. doi: 10.1186/s12859-020-3494-x (PMC7216538; doi:10.1186/s12859-020-3494-x)

**Example 1 Acute leukemia dataset**

Step 1 Prepare data and conduct exploratory analysis


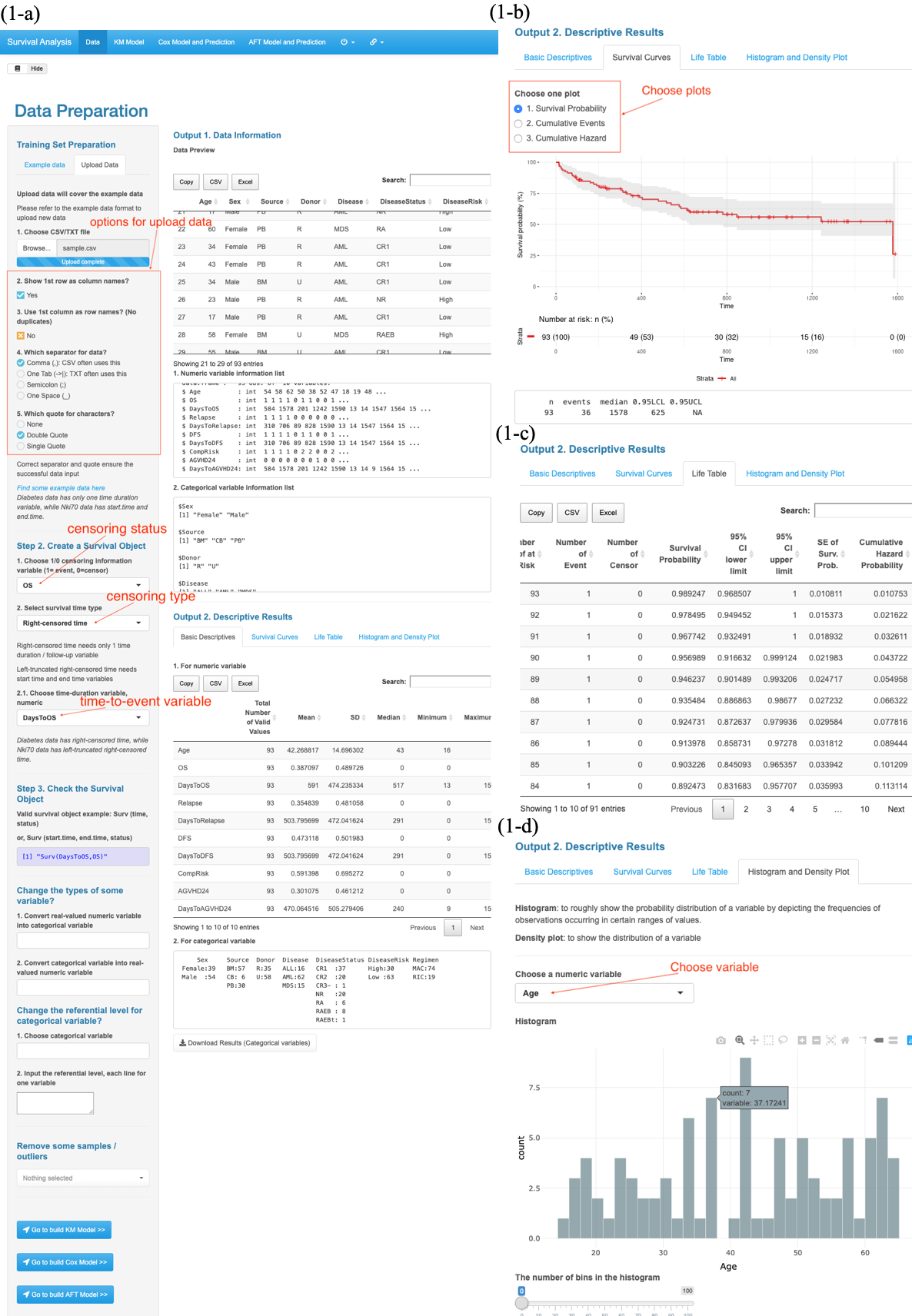


Step 2 Conduct log-rank test and generate results


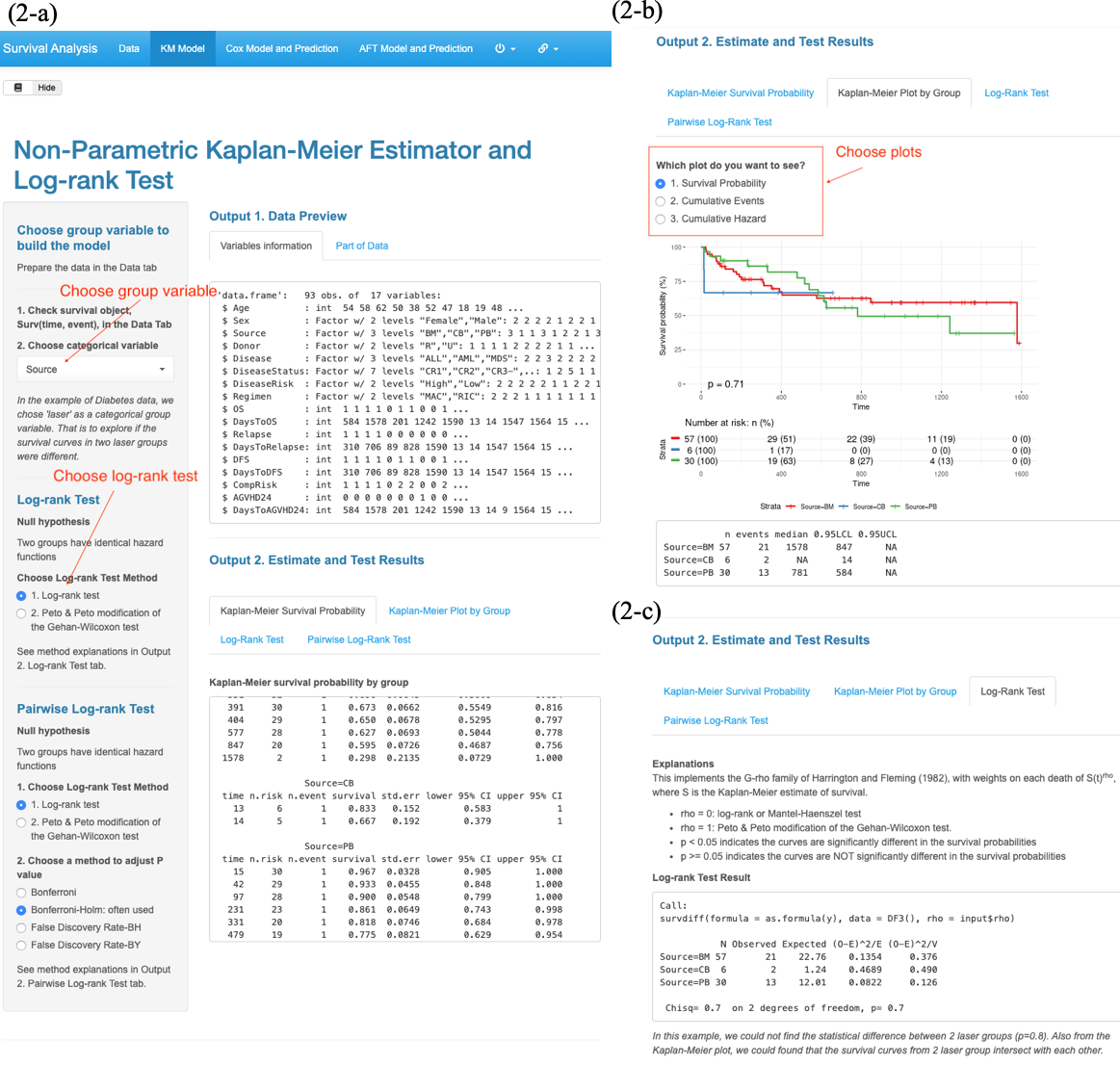


Step 3 Choose independent variables to build Cox regression and achieve results


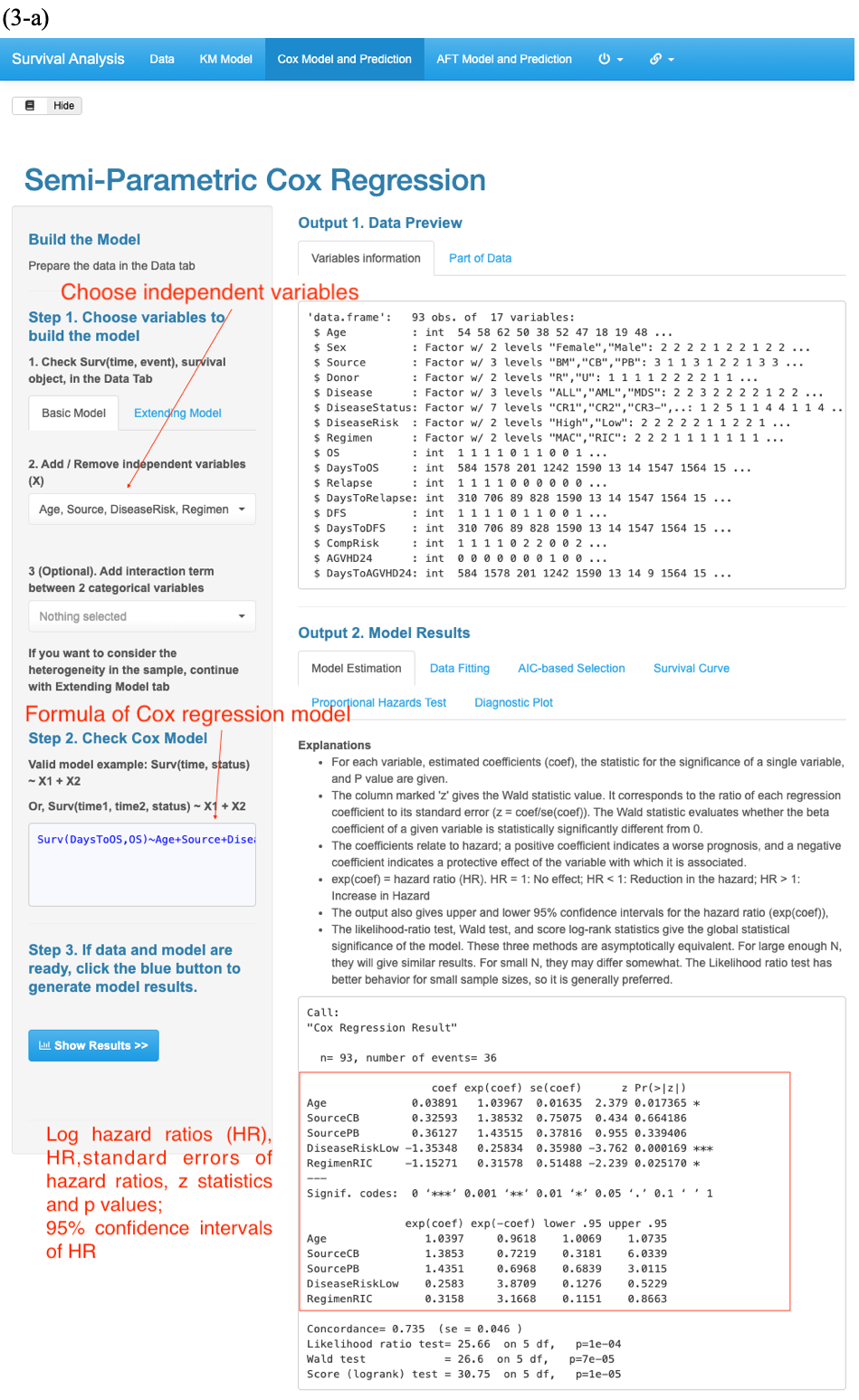


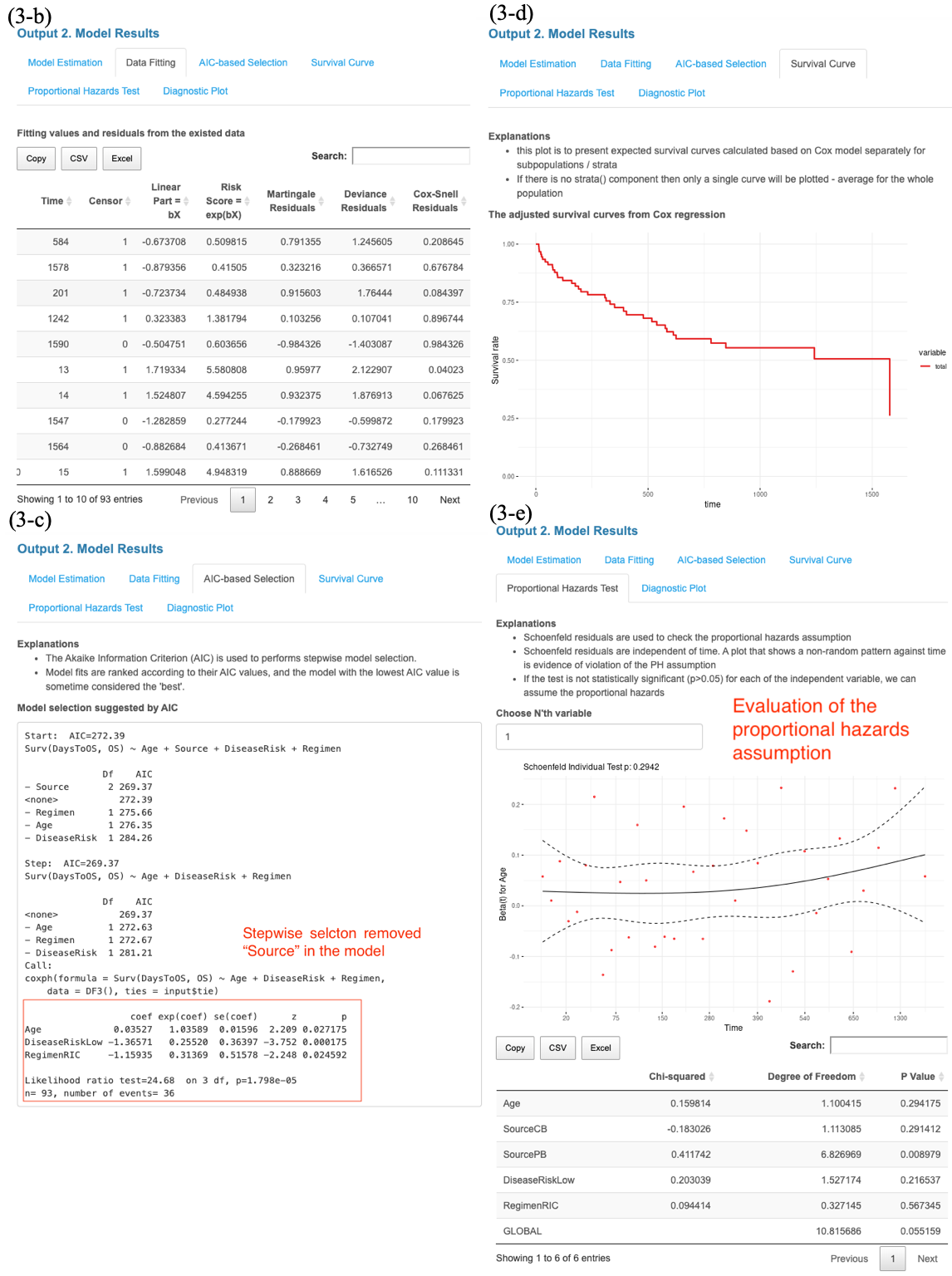


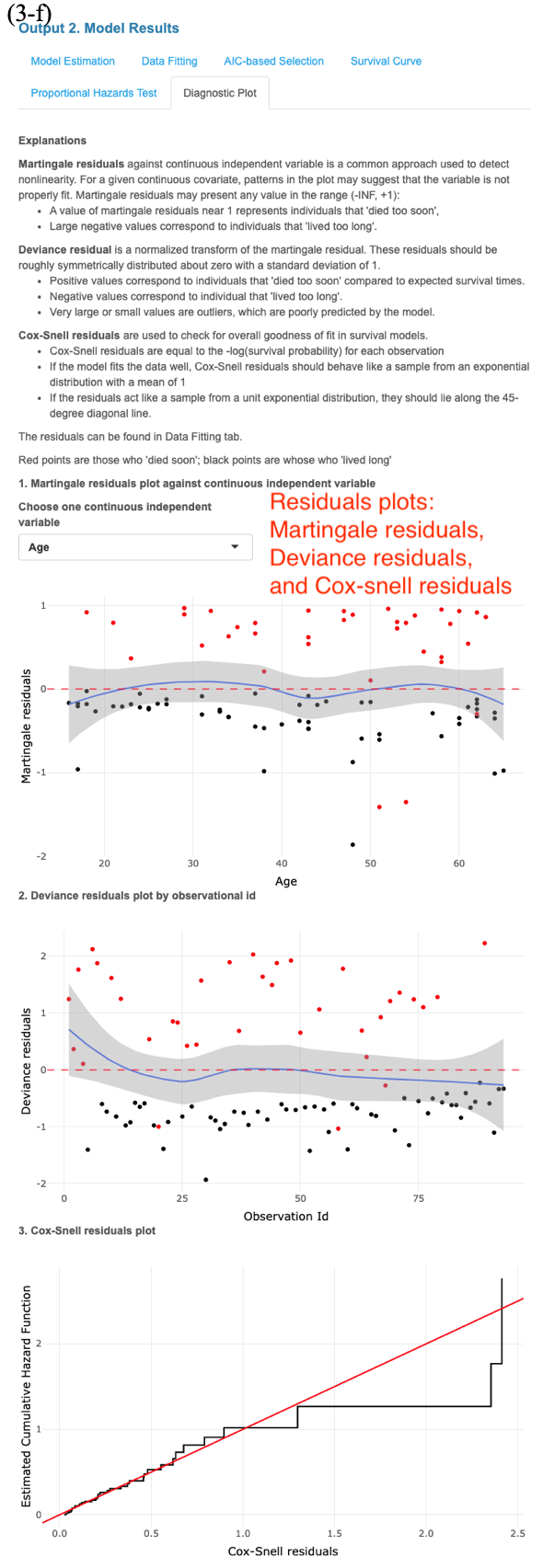


Step 4 Prediction in Cox regression with assessments of Brier Score and time-dependent AUC


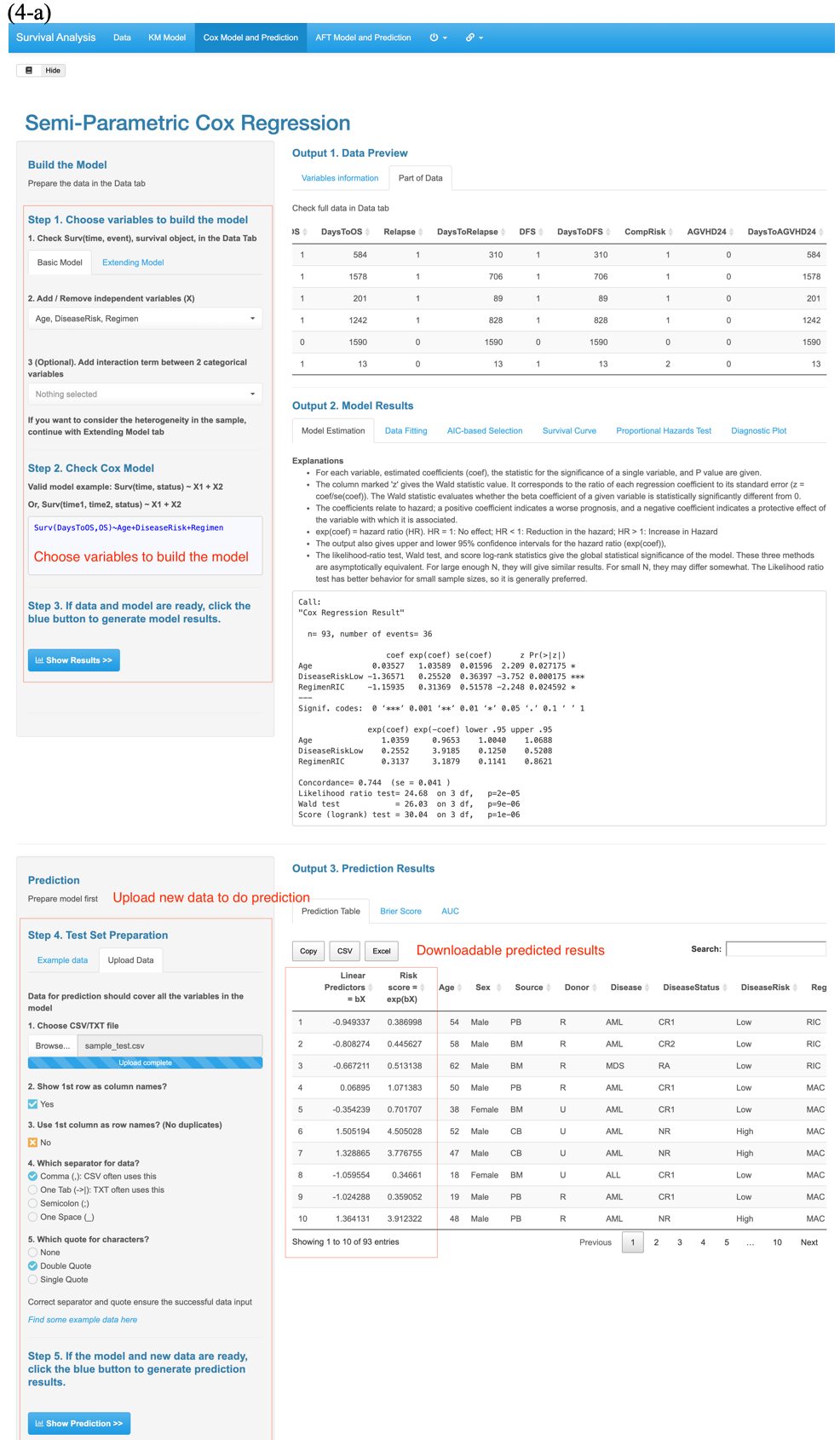


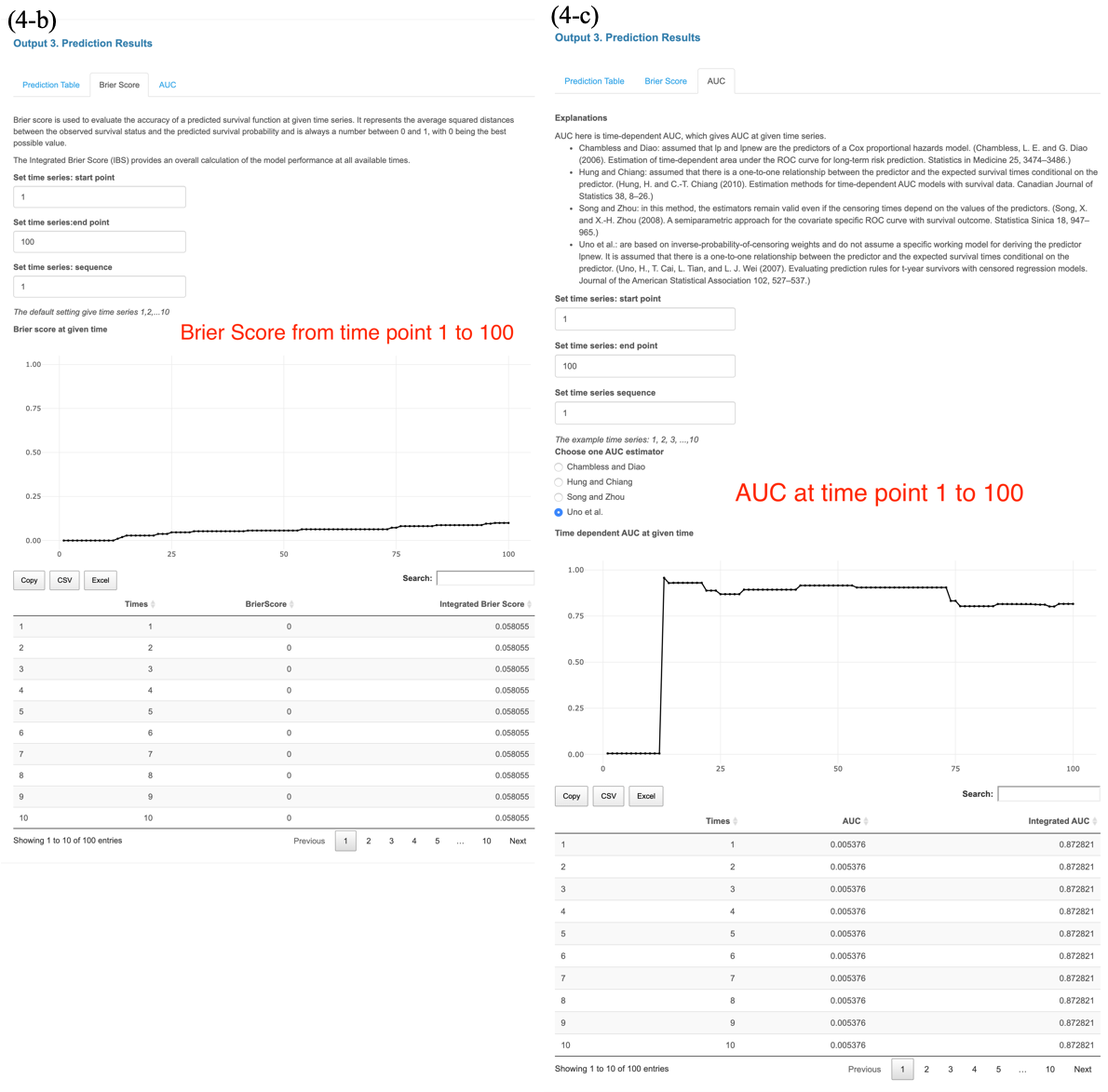


Step 5 Extending Cox regression to analyze the heterogeneity from relapse


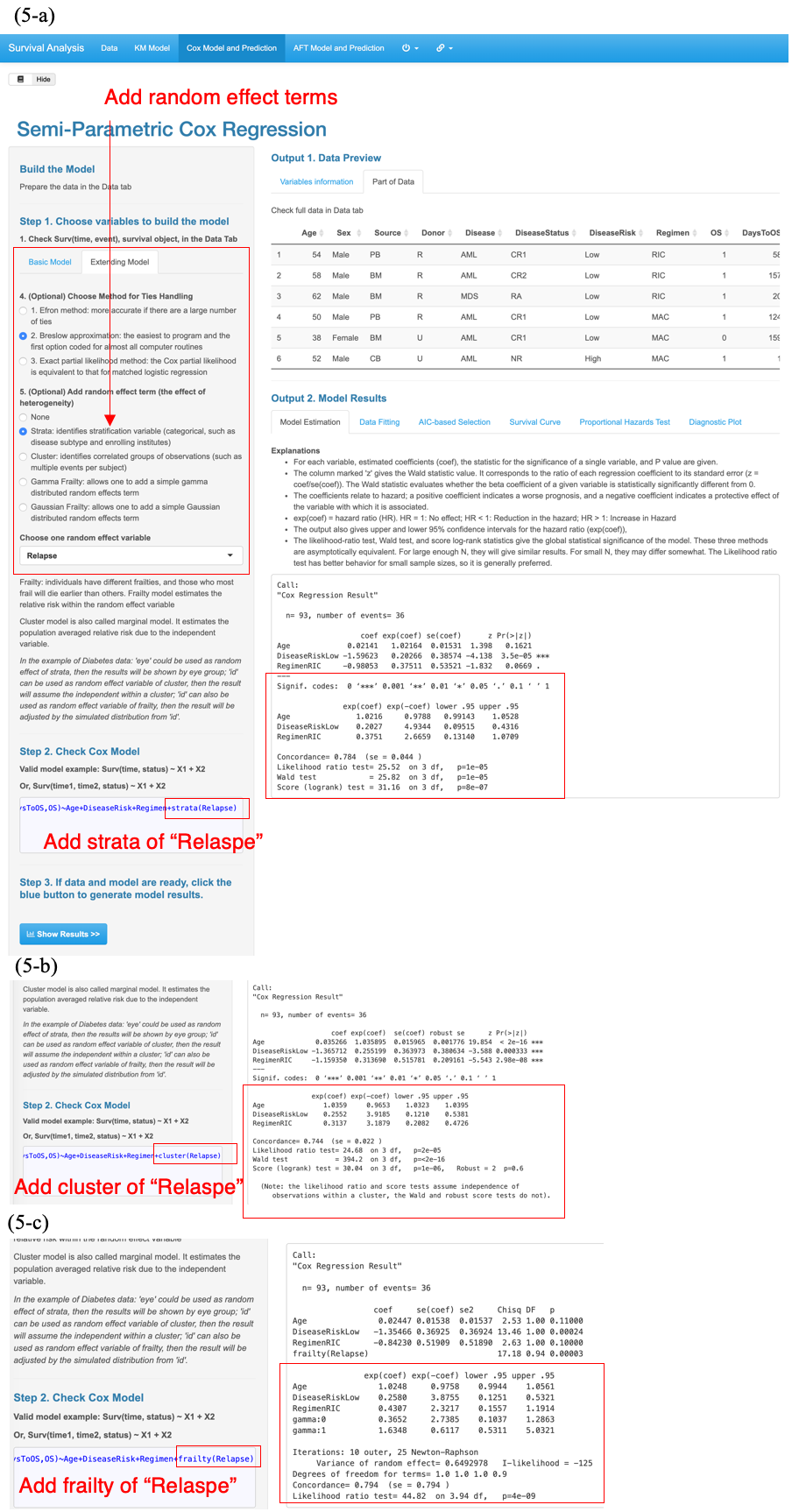


**Example 2 Yeast cell cycle dataset**

Step 1 Data preparation and exploratory analysis


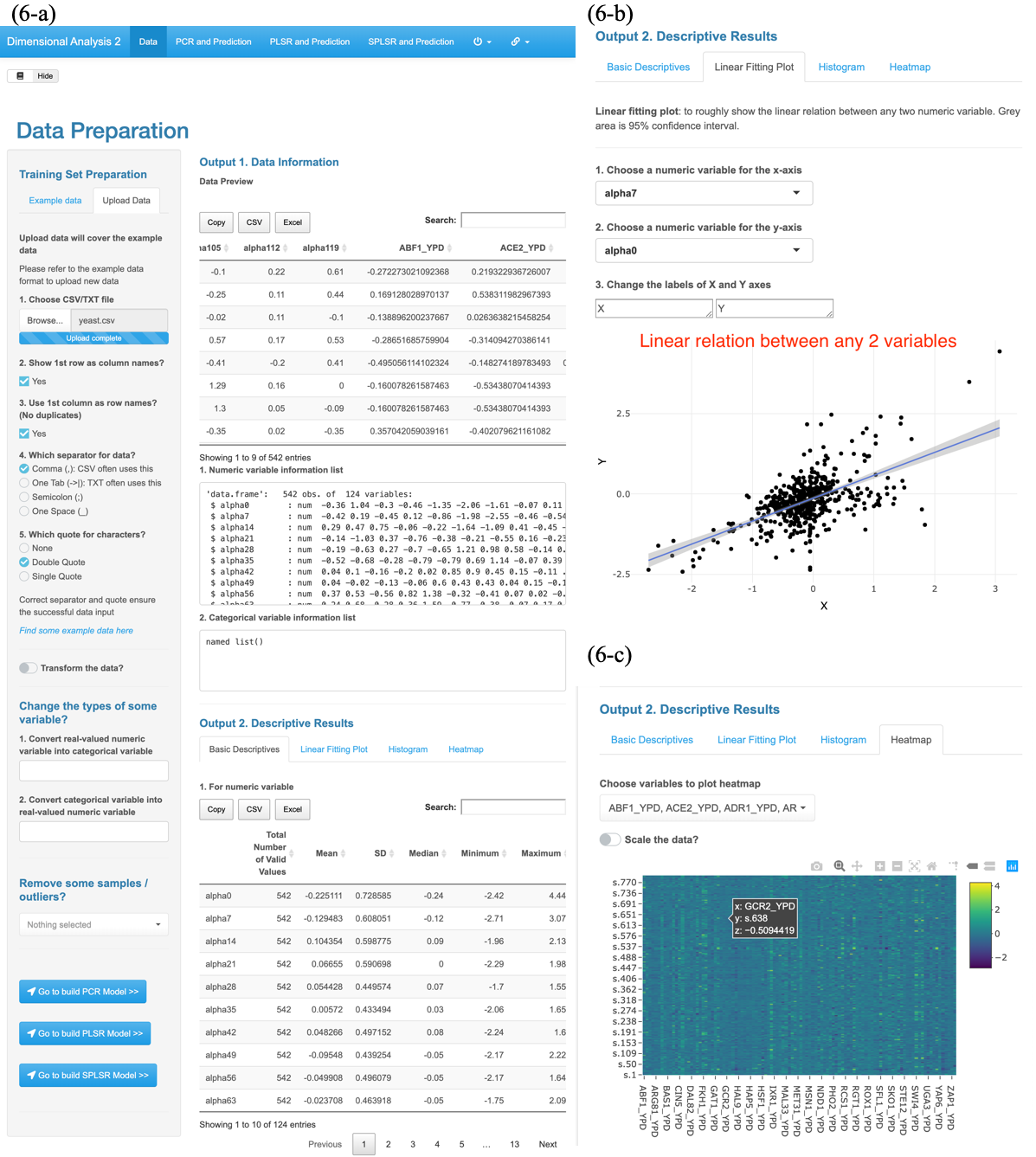


Step 2 Choose dependent and independent variables in multivariate SPLS-R and generate results


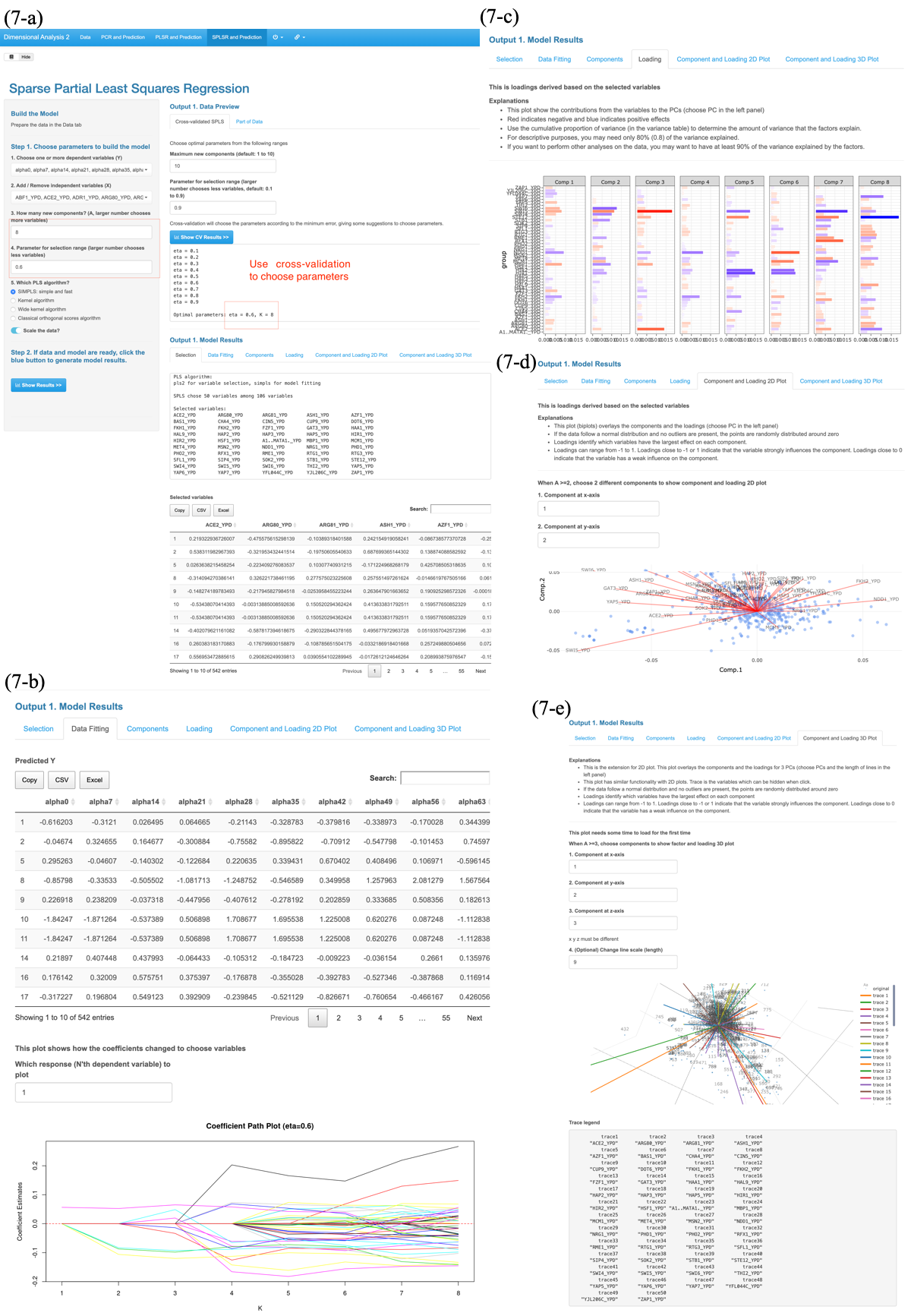


Step 3 Prediction in SPLS-R


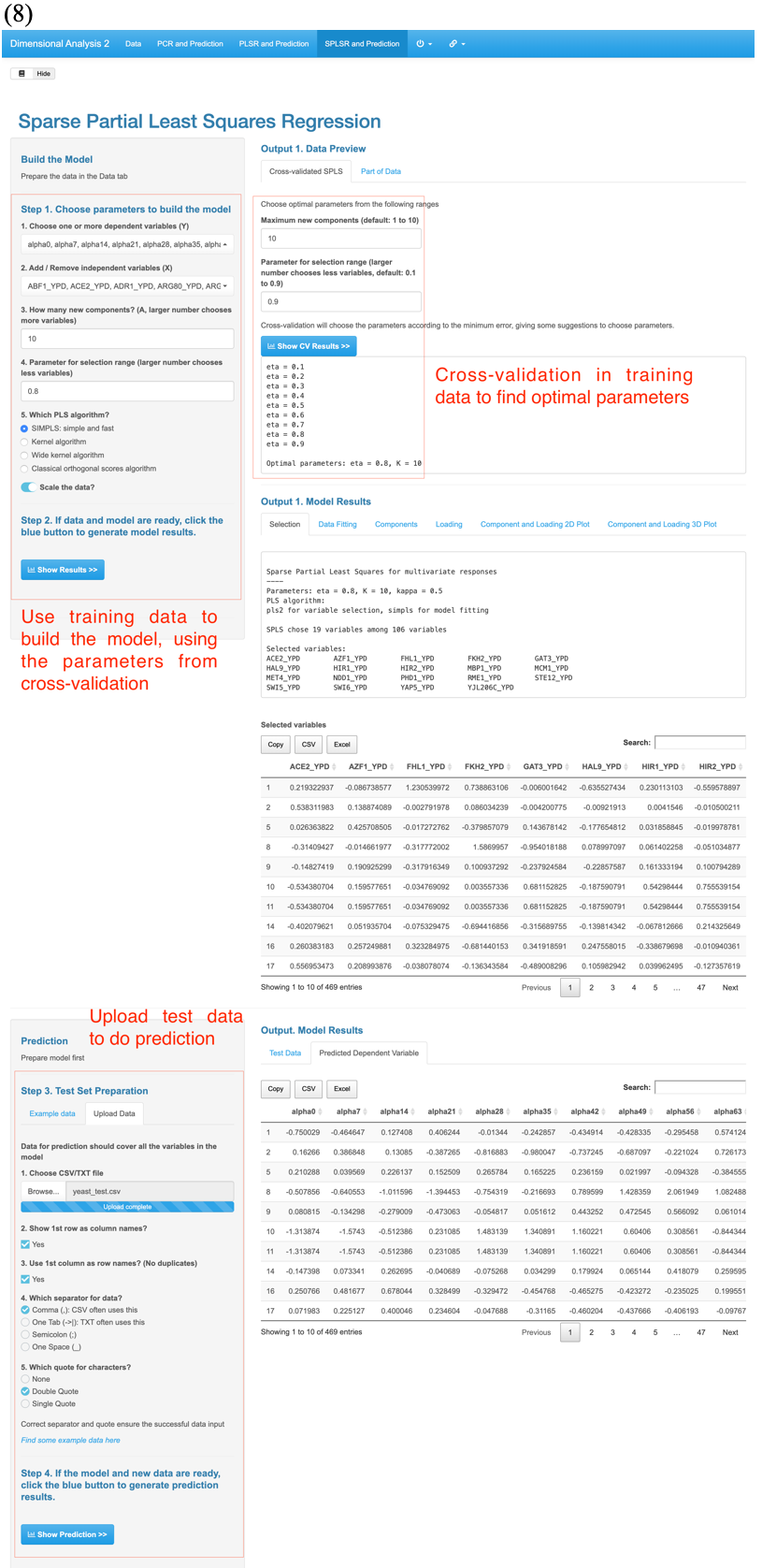

Supplement: Supplementary file 1 — Additional file 1: AF_table1.docx Graphic user interfaces and statistical methods in MEPHAS; AF_table2.docx Comparison of methods in MEPHAS with EZR, FSFS, and Radiant; AF_list.docx R packages used in MEPHAS; AF_result.docx The results in Example 1 and Example 2. [file 12859_2020_3494_MOESM1_ESM.zip › AF_result.docx]
