# Supplementary material for: MEPHAS: an interactive graphical user interface for medical and pharmaceutical statistical analysis with R and Shiny
Source: BMC Bioinformatics. 2020 May 11;21:183. doi: 10.1186/s12859-020-3494-x (PMC7216538; doi:10.1186/s12859-020-3494-x)
Supplement: Supplementary file 1 — Additional file 1: AF_table1.docx Graphic user interfaces and statistical methods in MEPHAS; AF_table2.docx Comparison of methods in MEPHAS with EZR, FSFS, and Radiant; AF_list.docx R packages used in MEPHAS; AF_result.docx The results in Example 1 and Example 2. [file 12859_2020_3494_MOESM1_ESM.zip › AF_table1.docx]

**Table 1 Graphic user interfaces and statistical methods in MEPHAS**

| **Categories of statistics** | **Graphic user interfaces** | **Statistical methods** | **Statistical table** | **Statistical plot** |
| --- | --- | --- | --- | --- |
| Probability distribution | Continuous probability distribution | - Normal distribution - Exponential distribution - Gamma distribution - Student’s t distribution - Chi-square distribution - F distribution | - Probability calculated according to the input - Random number - Mean and SD of random number - Mean and SD of user data | - Probability density function (PDF) - Cumulative density function (CDF) - Histogram of random numbers - Density, histogram, and empirical CDF of user data |
|  | Discrete probability distribution | - Binomial distribution - Poisson distribution | - Probability calculated according to the input - Random number - Mean and SD of random number - Mean and SD of user data | - Probability mass function (PMF) - Cumulative mass function (CMF) - Histogram of random numbers - Histogram and empirical CDF of user data |

| **Categories of statistics** | **Graphic user interfaces** | **Statistical methods** | **Statistical table** | **Statistical plot** |
| --- | --- | --- | --- | --- |
| Hypothesis testing | Parametric T test for means | - One sample t-test - Independent two sample t-test - F test for variances - Welch t-test - Paired samples t-test | - Basic descriptive statistics - Test result | - Box plot - Mean and SD pot - Normal QQ plot - Histogram - Density plot |
|  | Non-parametric test for medians | - One sample Wilcoxon signed-rank test - Two samples Wilcoxon rank-sum test - Two paired samples Wilcoxon signed-rank test | - Basic descriptive statistics - Test result | - Box plot - Mean and SD pot - Histogram - Density plot |
|  | Test for Binomial Proportion | - One proportion Chi-square test and Exact binomial test - Two proportions Chi-square test - More than two proportions Chi-square test - Chi-square test for trend | - Data with marginal sum - Test result | - Pie chart for the proportion |
|  | Test for Contingency Table | Chi-square test  Fisher’s exact test  McNemar’s test for paired data  Kappa statistic  Mantel-Haenszel test  Cochran-Mantel-Haenszel test | - Contingency table with marginal sum - Contingency table with expected values - Contingency table with percentages in row, column, and total - Agreement table and weight table for Kappa statistic - Test result | - Bar plot of percentages |
|  | Analysis of variance | One-way ANOVA and multiple comparison  Two-way ANOVA and multiple comparison  Non-parametric Kruskal-Wallis one-way ANOVA and multiple comparison | - Basic descriptive statistics - ANOVA table - Pairwise P value table | - Box plot - Mean and SD plot - Mean plot (two-way ANOVA) - Marginal mean plot (two-way ANOVA) |

| **Categories of statistics** | **Graphic user interfaces** | **Statistical methods** | **Statistical table** | **Statistical plot** |
| --- | --- | --- | --- | --- |
| Regression model | Linear regression | Data preparation  Linear model and prediction | - Basic descriptive statistics - Model estimation - Fitting values and residuals - ANOVA table of linear regression - AIC based stepwise selection - Prediction on new data | - Linear fitting plot of two variables - Histogram and density plot of a certain variable - Normal QQ plot of the residuals - Residuals versus fitting plot - Scatter plot in 3D - Linear fitting plot on new predicted values |
|  | Logistic regression | Data preparation  Linear model and prediction | - Basic descriptive statistics - Model estimation - Fitting values and residuals - AIC based stepwise selection - ROC table - Prediction on new data - ROC table from new data | - Linear fitting plot of two variables - Histogram and density plot of a certain variable - ROC plot - ROC plot in prediction |
|  | Survival analysis | Data preparation  Kaplan-Meier estimation and log-rank test  Cox regression with prediction  Accelerated failure time (AFT) model and prediction | - Basic descriptive statistics - Life table - Kaplan-Meier estimated survival probability - Log-rank and pairwise log-rank test - Model estimation of Cox regression - Fitting values and residuals from Cox regression - AIC based stepwise selection - Proportional hazard tests - Prediction on new data - Brier score (Cox prediction) - Time-dependent AUC (Cox prediction) - Predicted survival probability of a certain sample (AFT prediction) | - Survival probability, cumulative events, or cumulative hazards curves in all - Survival probability, cumulative events, or cumulative hazards curves by group - Survival curves from Cox regression - Proportional hazard tests plot - Martingale residuals plot - Deviance residuals plot - Cox-snell residuals plot - Brier score plot - Time-dependent AUC plot - Predicted survival plot |

| **Categories of statistics** | **Graphic user interfaces** | **Statistical methods** | **Statistical table** | **Statistical plot** |
| --- | --- | --- | --- | --- |
| Dimensional analysis | Dimensional analysis 1 | Principal component analysis (PCA)  Exploratory factor analysis (EFA) | - Basic descriptive statistics - Correlation matrix - Parallel analysis - Components or factors - Loading and variance table | - Linear fitting plot of two variables - Histogram and density plot of a certain variable - Heatmap - Correlation plot - Scree plot of the optimal number of components - Components scatter plot - Loading bar plots - Component and loading 2D biplot - Component and loading 3D biplot - Factor relation plot |
|  | Dimensional analysis 2 | Principal component regression (PCR)  Partial least squares regression (PLS-R)  Sparse partial least squares regression (SPLS-R) | - Basic descriptive statistics - Models results - Fitting values and residuals - Coefficient - Component - Loading - Prediction on new data - Predicted components from new data | - Linear fitting plot of two variables - Histogram and density plot of a certain variable - Heatmap - Components scatter plot - Loading bar plots - Component and loading 2D biplot - Component and loading 3D biplot |
