# Supplementary material for: MEPHAS: an interactive graphical user interface for medical and pharmaceutical statistical analysis with R and Shiny
Source: BMC Bioinformatics. 2020 May 11;21:183. doi: 10.1186/s12859-020-3494-x (PMC7216538; doi:10.1186/s12859-020-3494-x)
Supplement: Supplementary file 1 — Additional file 1: AF_table1.docx Graphic user interfaces and statistical methods in MEPHAS; AF_table2.docx Comparison of methods in MEPHAS with EZR, FSFS, and Radiant; AF_list.docx R packages used in MEPHAS; AF_result.docx The results in Example 1 and Example 2. [file 12859_2020_3494_MOESM1_ESM.zip › AF_table2.docx]

**Table 2 Comparison of methods in MEPHAS with EZR, FSFS, and Radiant**

| **Categories of statistics** | **MEPHAS** | **EZR** | **Free Statistics and Forecasting Software (FSFS)** | **Radiant** |
| --- | --- | --- | --- | --- |
| Probability distribution | PDF, **CDF, random number** of   - Normal distribution - Exponential distribution - Gamma distribution - Student’s t distribution - Chi-square distribution - F distribution - Binomial distribution - **Poisson distribution** |  | - Random Number Generator - Normal Distribution - **Maximum likelihood fitting** - CDF Normal - **Random Number Generator - Log-Normal Distribution** - Area under Normal Density - **Skewness/Kurtosis Test** - Area under Binomial Density | PDF of   - **Uniform distribution** - Normal distribution - Exponential distribution - Gamma distribution - Student’s t distribution - Chi-square distribution - F distribution - Binomial distribution   **Central limit theorem** |

| Hypothesis testing | - One sample t-test - Independent two sample t-test - F test for variances - Welch t-test - Paired samples t-test - Basic descriptive statistics - One sample Wilcoxon signed-rank test - Two samples Wilcoxon rank-sum test - Two paired samples Wilcoxon signed-rank test - One proportion Chi-square test and Exact binomial test - Two proportions Chi-square test - More than two proportions Chi-square test - Chi-square test for trend - Chi-square test - **Fisher’s exact test** - McNemar’s test for paired data - **Kappa statistic** - Mantel-Haenszel test - **Cochran-Mantel-Haenszel test** - One-way ANOVA and **multiple comparison** - Two-way ANOVA and **multiple comparison** - Non-parametric one-way ANOVA and **multiple comparison** | | | - Numerical summaries - **Smirnov-Grubbs test for outliers** - **Kolmogorov-Smimov test for normal distribution** - Confidence interval for a mean - Single-sample t-test - Two-variances F-test - Two-sample t-test - Paired t-test - **Test for Pearson's correlation** - Mann-Whitney U test - Wilcoxon's signed rank test - **Friedman test** - **Jonckheere-Terpstra test** - **Spearman's rank correlation test** - Frequency distributions/cr Confidence interval for a proportion - One sample proportion test - Confidence interval for a difference between two proportions - Confidence interval for a ratio of two proportions - Compare two proportions (Fisher's exact test and Chi-square test) - **Compare proportions of more than two paired samples (Cochran Q test)** - Cochran-Armitage test for trend in proportions - Compare proportions of two paired samples (McNemar test) - Mantel-Haenzel test for matched proportions - One-way ANOVA - **Repeated-measures ANOVA** - **Multi-way ANOVA** - **ANCOVA** - Kruskal-Wallis test - **Bartlett's test** | - Testing Mean - Two Sample Tests about the Mean - Testing Variance - **D'Agostino skewness test, Anscombe-Glynn kurtosis test, Jarque-Bera Normality Test (against normality)** - **Kendall tau Correlation Matrix** - **Compare Correlations** - Testing Population Proportion - Chi-Squared Tests - 1-way ANOVA - 2-way ANOVA | | - Single mean - Compare means - Single mean - Compare means - Single proportion - Compare proportions - Goodness-of-fit - Cross-tab - Correlation | |
| --- | --- | --- | --- | --- | --- | --- | --- | --- |
| **Categories of statistics** | | **MEPHAS** | **EZR** | | | **Free Statistics and Forecasting Software** | | **Radiant** |
| Regression model | | Linear model  **Prediction** | - Linear regression | | | - Simple regression - Multiple regression | | - Linear regression |
|  |  | Linear model  **Prediction** | - Logistic regression - **Conditional logistic regression for matched-pair analysis** | | | - Logistic regression | | - Logistic regression - **Multinomial logistic regression** |
|  |  | Righted-censored time and **left-truncated and right-censored time**   - Kaplan-Meier estimation and log-rank test - Cox regression - **Cox regression with random effect terms** - **Prediction in Cox regression** - **Accelerated failure time (AFT) model** - **Prediction in AFT model** | - Kaplan-Meier survival curve and log-rank test - **Log-rank trend test** - Cox proportional hazard regression - **Cox proportional hazard regression with time-dependent covariate** - **Cumulative incidence of competing events and Gray test** - **Fine-Gray proportional hazard regression for competing events** - Stratified Cox proportional hazard regression for matched-pair analysis | | | - NA | | - NA |

| **Categories of statistics** | **MEPHAS** | **EZR** | **Free Statistics and Forecasting Software** | **Radiant** |
| --- | --- | --- | --- | --- |
| Dimensional analysis | Principal component analysis (PCA)  Exploratory factor analysis (EFA)  **Principal component regression (PCR)**  **Prediction of PCR**  **Partial least squares regression (PLS-R)**  **Prediction of PLS-R**  **Sparse partial least squares regression (SPLS-R)**  **Prediction of SPLS-R** | - Principal-components analysis - Factor analysis | - **Path Models by Partial Least Squares** | - **Pre-factor analysis** - Factor analysis |

| **Categories of statistics** | **MEPHAS** | **EZR** | **Free Statistics and Forecasting Software** | **Radiant** |
| --- | --- | --- | --- | --- |
| Others not available in MEPHAS | NA | - **Meta-analysis and meta-regression test** - **Sample size and power calculation** - **Cluster methods** | - **Regression tree** - **Time Series Analysis (R modules)** | - **Sample size** - **Classification and regression tree** - **Naïve bayes** - **Neural network** - **Decision analysis** - **Dissimilarity/Attributts** - **Cluster methods** - **Conjoint** |

Note: methods in bold are those not given in the other software;

the methods list of EZR is found http://www.jichi.ac.jp/saitama-sct/SaitamaHP.files/functions.html
